# Genome-wide association analysis revealed genetic variation and candidate genes associated with the yield traits of upland cotton under drought conditions

**DOI:** 10.3389/fpls.2023.1135302

**Published:** 2023-04-12

**Authors:** Fenglei Sun, Jun Ma, Weijun Shi, Yanlong Yang

**Affiliations:** ^1^ State Key Laboratory of Cotton Biology, Institute of Cotton Research of the Chinese Academy of Agricultural Sciences, Anyang, China; ^2^ Hainan Yazhou Bay Seed Laboratory, Sanya, Hainan, China; ^3^ Research Institute of Economic Crops, Xinjiang Academy of Agricultural Sciences, Urumqi, China

**Keywords:** upland cotton, drought tolerance, genome-wide associated study, SNPs, candidate genes

## Abstract

Drought is one of the major abiotic stresses seriously affecting cotton yield. At present, the main cotton-producing areas in China are primarily arid and semiarid regions. Therefore, the identification of molecular markers and genes associated with cotton yield traits under drought conditions is of great importance for stabilize cotton yield under such conditions. In this study, resequencing data were used to conduct a genome-wide association study (GWAS) on 8 traits of 150 cotton germplasms. Under drought stress, 18 SNPs were significantly correlated with yield traits (single-boll weight (SBW) and seed (SC)), and 8 SNPs were identified as significantly correlated with effective fruit shoot number (EFBN) traits (a trait that is positively correlated with yield). Finally, a total of 15 candidate genes were screened. The combined results of the GWAS and transcriptome data analysis showed that four genes were highly expressed after drought stress, and these genes had significantly increased expression at 10, 15 and 25 DPA of fiber development. qRT-PCR was performed on two samples with drought tolerance extremes (drought-resistant Xinluzao 45 and drought-sensitive Xinluzao 26), revealing that three of the genes had the same differential expression pattern. This study provides a theoretical basis for the genetic analysis of cotton yield traits under drought stress, and provides gene resources for improved breeding of cotton yield traits under drought stress.

## 1 Introduction

As an important cash crop in China, cotton plays a major role in domestic economic development. However, with the increase in extreme climatic events in recent years and the decrease in water resources, abiotic stress (drought) has become increasingly serious (Sun et al., 2017; Sun et al., 2021). Drought, in response to global warming, is becoming more prominent and serious, and it has become a focus in climate change research (Cattivelli et al., 2008). Some arid or semiarid areas are experiencing annual decreases in precipitation and fresh water resources. Even in areas with relatively sufficient water resources, the effect of extreme climate on local precipitation is noticeable, and it is particularly obvious in China (Wang et al., 2017). Cotton cultivation in Chian is located mainly in Xinjiang, the Yangtze River Basin and the Huang-Huai-Hai region (Huang et al., 2017). According to data from the National Bureau of Statistics, the sown area of cotton in Xinjiang in 2021 was 37.5926 million Mus, accounting for 82.76% of the national planting area (Ding et al., 2021). Xinjiang is located in an arid and semiarid region with little precipitation, and agricultural water consumption accounts for approximately 94% of total water consumption (Xiao, 2020). The cotton planting area in Xinjiang accounts for approximately 45% of the total sown area of crops in Xinjiang. With the frequent occurrence of extreme weather resulting from such as climate warming, lack of fresh water resources and high temperature, drought has become an important factor restricting cotton production in Xinjiang; furthermore, drought stress can affect the yield and quality of cotton by changing its metabolic activities and biological functions.

To date, many genes associated with drought tolerance have been identified. Li et al. (2019) studied the drought resistance of 316 upland cotton germplasms at the seedling stage by GWAS and identified *WRKY70*, *GhCIPK6*, *SnRK2.6* and *NET1A* as genes induced by drought stress. In rice, Sun et al. found that *DROT1* can improve drought tolerance, mainly by regulating the cell wall fiber content and crystal structure in microtubule tissues to enhance drought resistance (Sun et al., 2022). Du et al. found that *TaERF87* (ethylene response factor (ERF)) could interact with *TaAKS1* to enhance the expression of *TaP5CS1* and *TaP5CR1*, thus improving proline synthesis and drought resistance in wheat (Du et al., 2022). Through a GWAS in maize, it was found that three SNP mutations in *ZmSRO1d* significantly increased the reactive oxygen species (ROS) content of guard cells, promoted stomatal closure, enhanced drought resistance, and increased the yield of overexpressed *ZMSRO1D-R* by 60% compared with that in the control (Gao et al., 2022). Although these identified drought tolerance genes are associated with different traits, they can all improve the drought tolerance of related crops.

Previously, most drought-resistant quantitative trait loci (QTLs) were identified in genetic populations through simple sequence repeat (SSR) markers (Sang et al., 2017), but with the advancement of sequencing technology and the completion of cotton genome sequencing, GWAS has become an important analytical tool (Li et al., 2019). Some QTL sites associated with drought tolerance traits have also been identified by the GWAS approach. Saleem et al. (2015) used 524 SSR markers to perform linkage analysis of F_2_ populations of drought-tolerant (B-557) and drought-resistant (FH-1000) varieties and detected 22 drought-related QTLs. These included two QTLs related to water content and QTLs on chromosome 23 that were associated with leaf water loss. Shukla et al. (2021) performed drought tolerance genetic mapping and QTL analysis of drought-tolerant (AS2) and drought-susceptible (MCU13) terrestrial cotton recombinant inbred line (RIL) populations based on genotyping by sequencing (GBS) and SSRs and identified 19 QTLs associated wtih field drought-tolerance traits, with 3 QTLs on chromosome 8 related to relative water content. Hou et al. (2018) genotyped 319 land cotton accessions through a high-density CottonSNP80K array, found that 20 quantitative trait nucleotides (QTNs) distributed on 16 chromosomes were associated with 6 drought resistance traits, and finally identified two candidate genes related to soluble sugar content and one gene related to root dry matter and hypocotyl length. Abdelraheem et al. (2020a); Abdelraheem et al. (2021) constructed a multiparent advanced generation intercross (MAGIC) population with 11 upland cotton accessions as parents and performed a GWAS of drought resistance traits in 550 strains. A total of 23 and 20 QTLs were detected under normal and drought-resistant treatment conditions, respectively. A GWAS performed on 376 upland cotton seedlings in the United States to investigate drought tolerance revealed 13 QTL clusters at 11 sites. Based on 372 strains derived from MAGIC populations with 8 upland cotton accessions as parents, Huang et al. (2021) used specific locus amplified fragment sequencing (SLAF-seq) to map genome-wide associations, and found that 177 SNPs were significantly associated with 9 stable agronomic traits in multiple environments, and 8 candidate genes with known functions were identified. Ul-Allah et al. (2021) reported that the effects of drought stress on cotton fiber development can lead to a yield loss of approximately 45%. Abdelraheem et al. (2020b) showed that water deficit during flowering can lead to a decrease in cotton fiber strength, an increase in staple fiber content, and a decrease in quality. Most association analyses of drought resistance in cotton populations were based on SSR markers, GBS and gene chips, and there are few reports that use resequencing to locate drought resistance sites. Moreover, studies on the localization of key trait QTLs in cotton have focused mainly on fiber quality, while there have been relatively few studies on the localization of QTLs associated with yield traits under drought conditions.

Although some genes or QTLs associated with yield traits have been identified in genetic populations and natural populations, effective analysis of the genetic basis of yield traits is still incomplete. Therefore, in this study, we collected phenotypic data from 150 upland cotton cultivars with large yield differences in the Shihezi and Korla areas of Xinjiang. Furthermore, we analyzed and explored the genetic loci and key candidate genes related to yield under drought conditions through the GWAS approach, which laid a foundation for studying the molecular mechanism underlying cotton drought resistance and the genetic improvement of cotton.

## 2 Materials and methods

### 2.1 Plant material and drought stress treatment

A total of 152 land cotton germplasms (**Supplementary Table S1**) were collected, all of which are cultivars grown in Northwest China, and were collected and preserved by the Xinjiang Academy of Agricultural Sciences. In 2019 and 2020, 152 land cotton germplasms were planted in 2 natural environments, namely Shihezi (85.94°E, 44.27°N) in 2019 and Korla (86.06°E, 35.05°N) in 2020. All accessions were planted following a random complete block design (RCBD) with two replicates per environment and two rows per replicate. In both Korla and Shihezi, the row length was 2 meters, the row spacing was 66 + 10 cm (width/narrow), and the plant distance was 10 cm. The conditions for drought stress treatment were achieved by artificial water control. The treatment was mainly applied at the flowering and boll stages, with irrigation stopped in the drought stress treatment group and continued in the control group. During the boll opening period, irrigation was reinitiated in the drought stress treatment followed by normal irrigation.

### 2.2 Phenotypic data collection and analysis

After cotton maturation, three yield-related traits and five agronomic traits, namely seed cotton (SC), single boll weight (SBW), lint cotton (LC), plant height (PH), fruit branch number (FBN), effective fruit branch number (EFBN), boll number (BN) and effective boll number (EBN), were measured under each environmental condition to analyze phenotypic changes in cotton. For each variety, 10 plants were randomly selected from the middle of each row. The five agronomic traits were measured, with ten biological replicates for each germplasm. Twenty mature bolls were randomly harvested from the middle part of the cotton plant and weighed (BW), with 2 bolls per plant. After ginning, the LC and SC were weighed and counted separately. The survey method followed the Specification for the Description of Cotton Germplasm Resources and Data Standard guidelines (Du and Zhou, 2005). In this study, data for eight traits (including five agronomic traits and three yield traits) in two environments were statistically analyzed. SPSS 25.0 was used for descriptive statistical analysis of all traits as well as analysis of variance. Correlation analysis of all traits in the cotton panel across different environments was performed in R software. Since the seedlings of 2 accessions were not sufficient for phenotypic studies, we used data from eight phenotypic traits of 150 accessions for the subsequent GWAS.

### 2.3 Genotypic data analysis

Young leaves were collected from plants of the 150 accessions, and genomic DNA was extracted to construct paired end-sequencing libraries for resequencing with 10× genome coverage using the HiSeq 2000 platform (Illumina, Inc., San Diego, California, USA) (He et al., 2021). Clean reads from 150 germplasms were matched with the *Gossypium hirsutum* reference genome TM-1 [CRI v1 (Yang et al., 2019)] using BWA version 0.7.10. After alignment, SNP calling was performed at the population scale with a unified genotype approach using Genomic Analysis Toolkit (GATK, v3.1) (McKenna et al., 2010). Subsequently, high-quality SNPs with a reserved minor allele frequency (MAF) greater than 0.05 were used for further analysis.

### 2.4 LD analysis, population structure, haplotype analysis and clustering

Population linkage disequilibrium (LD) was analyzed by PopLDdecay software (Zhang et al., 2019), and r^2^ was calculated for SNPs within a 1 Mb window. Population structure was analyzed by the Admixture 1.3 program, which was run 1000 times for K values of 2-10 to generate admixture ratios. Then, the optimal value of K was determined by cross-validation (CV) scores and log-likelihood estimates. Haplotypes were detected and analyzed by software such as IGV (Helga et al., 2012), Tassel (Bradbury et al., 2007), Figtree (Rambaut, 2009), and R. First, strong and continuous SNP regions were identified by IGV software, and these regions were named target SNP intervals. Then, Tassel software was used to perform LD segment analysis and to identify numerical genotypes of target SNP intervals. The digital genotypes of “Minor”, “Major” and “Hereozygous” were filled with the color scale function in Excel, and haplotype classification was performed according to the color change in the target SNP interval. To construct a phylogenetic tree, the neighbor-joining (NJ) method in Tree Best (v1.9.2) software was used, and the tree was visually edited by Figtree software (Vilella et al., 2009).

### 2.5 Genome-wide association studies

To ensure the accuracy of the results, SNPs with a missing genotype frequency greater than 0.05 or a MAF less than 0.05 were filtered without imputation. A total of 2,499,987 SNPs were identified in the association panel for the final 150 samples, and the SNPs for the entire genome were viewed using the sliding window method (defaults of 50 bp window size and 10 bp steps). A GWAS between SNPs and traits was performed using Efficient Mixed Model Association Acceleration (EMMAX) software (Kang et al., 2010) and Fixed and random model Circulating Probability Unification (FarmCPU) models, where the threshold for association detection was set to -log(1/N) (where N is a valid value for the SNP label) (Li et al., 2012; Li et al., 2019).

### 2.6 Prediction of candidate genes and qRT-PCR

In this study, the upstream and downstream 200-600 kb windows of the genomes were scanned to screen for genes near each significant marker-trait association. The screened genes were identified and analyzed, and information on the annotated genes in upland cotton was downloaded from CottonFGD (https://cottonfgd.org) to search for additional potential annotated genes. Transcriptome data of ovule (3, 0, 1, 3, 5, 10, 15, 20, and 25DPA) and fiber tissues (10, 15, 20, and 25DPA) were also downloaded from the NCBI Sequence Read Archive collection PRJNA490626 (Hu et al., 2019). The role of potential candidate genes in responding to abiotic stresses, especially drought, was further analyzed by consulting the relevant literature.

qRT-PCR was used to analyze and screen the relative expression levels of candidate genes related to drought tolerance traits in cotton after drought stress. Leaf samples were collected from drought-tolerant Xinluzao 45 and drought-sensitive Xinluzao 26 (Sun et al., 2021), and total RNA was extracted by a TRIzol kit (Thermo Fisher, Beijing, China). cDNA was synthesized by a one-step RT-PCR kit (Novoprotein Scientific, China). The *GhUBQ7* gene was used as an internal control for data normalization. Gene expression was calculated by the 2^−ΔΔCt^ method (Tanino et al., 2017). The primers selected for this experiment are shown in **Supplementary Table S2**.

## 3 Results

### 3.1 Analysis of variations in yield and agronomic traits

The phenotypic variation in drought tolerance in 150 upland cotton materials was analyzed by measuring 8 drought tolerance related traits, including PH, FBN, EFBN, BN, EBN, SC, LC and SBW. There were differences in all traits between control and drought treatment (**Supplementary Table S3** and **Supplementary Figure S1**). In the control and the two-year average, the PH of the different materials ranged from 52.4-157.2 cm, the FBN ranged from 5.82-15, the EFBN ranged from 5.2-13, the BN ranged from 3.5-15.1, and the EBN ranged from 3.8-14.1 (**Supplementary Table S3**). SC ranged from 103.15-181.45 g, LC ranged from 35.27-65.03 g, and SBW ranged from 5.16-9.08 (**Supplementary Table S3**). After exposure to drought stress, averaged over 2 years, the PH of the different materials ranged from 33.02-78.8 cm, the FBN ranged from 3.69-11.1, the EFBN ranged from 3.8-9.05, the BN ranged from 1.34-10, and the EBN ranged from 1.09-4.45. SC ranged from 79.67-138.5 g, LC ranged from 27.9-57.95g, and SBW ranged from 3.98-6.93 g (**Supplementary Table S3**). Thus, the eight phenotypic traits were affected by drought stress in all samples, with PH, FBN, EFBN, BN, EBN, SC, LC and SBW decreasing by 24.05, 24.89, 21.54, 44.95, 68.05, 20.20, 21.29 and 20.20%, respectively, under drought stress (**Supplementary Table S3**). Based on the data collected over two years, the coefficient of variation of BN was higher (25.39 and 47.46% for the control and drought stress treatments, respectively), while that of SC was lower (9.17 and 9.59% for the control and drought stress treatments, respectively) (**Supplementary Table S3**). Except for the control of individual traits, the phenotypic differences of all traits were extremely significant or significant (p<0.05) when considering the single year and two-year averages, and the frequency distribution of all traits was consistent with a normal distribution (**Supplementary Figure S2**). The correlation analysis results for the normal and drought stress treatments in 2019 and 2020 are shown in **Supplementary Table S4**. In the control, there were extremely significant positive correlations between the five agronomic traits (p<0.01). Among the yield component traits, LC and SBW had extremely significant positive correlations with FBN, BN, EBN, EFBN, EBN and SC. However, after drought stress treatment, there were extremely significant positive correlations between all the traits except EFBN and SC; in particular, the yield trait SBW was positively correlated with the other traits (p<0.01).

### 3.2 Group characteristic analysis and LD analysis

**Figure 1:**
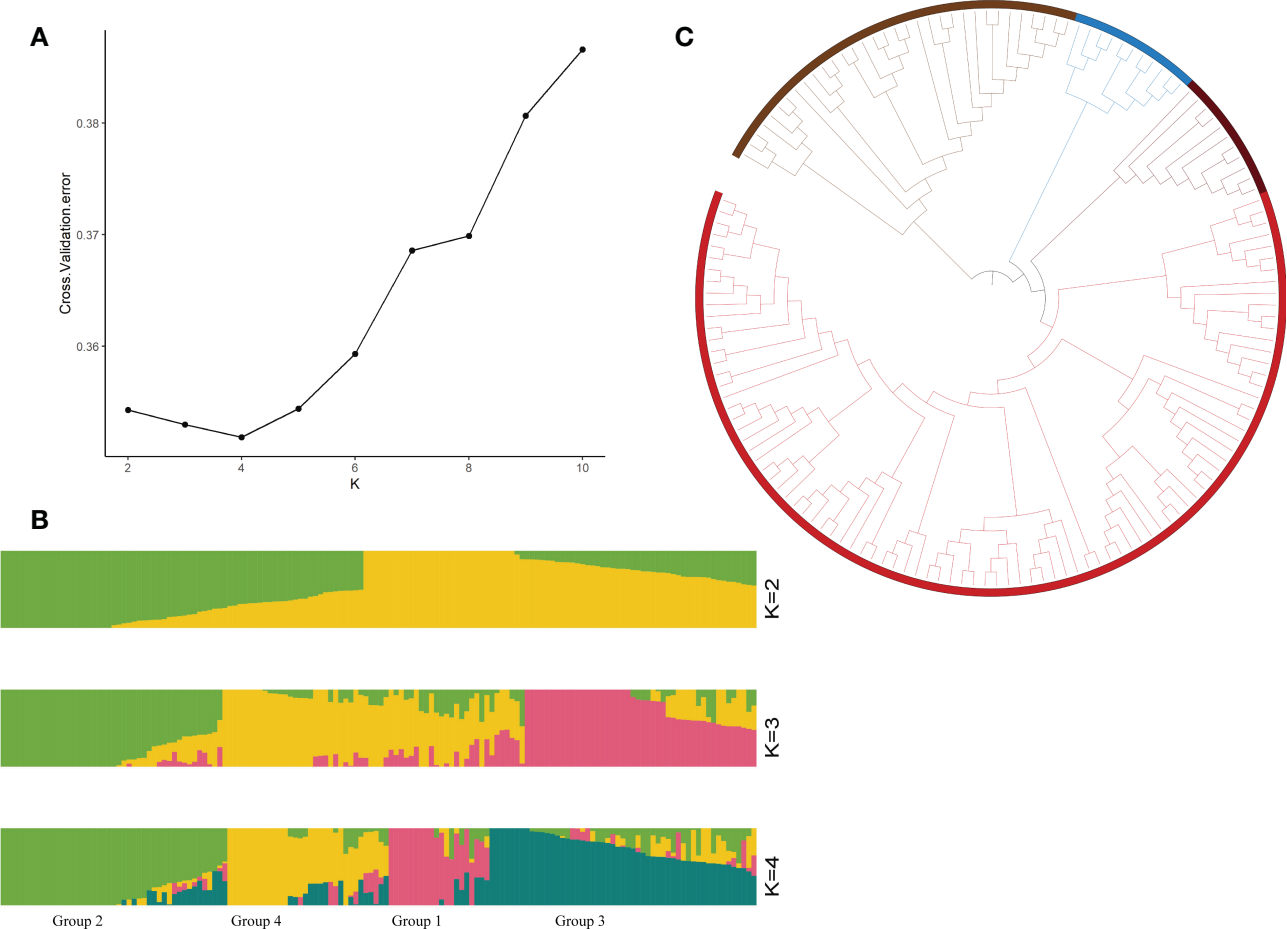
Genotyping analyses of 150 cotton germplasms. **(A)** Cross-validation diagram of the SNP dataset, **(B)** population structure analyzed by STRUCTURE at K=2, 3, and 4 (Group 1: High drought resistance, Group 2: Medium drought resistance, Group 3: Drought tolerance, Group 4: Drought sensitivity), and **(C)** phylogenetic tree of the population.

**Figure 2:**
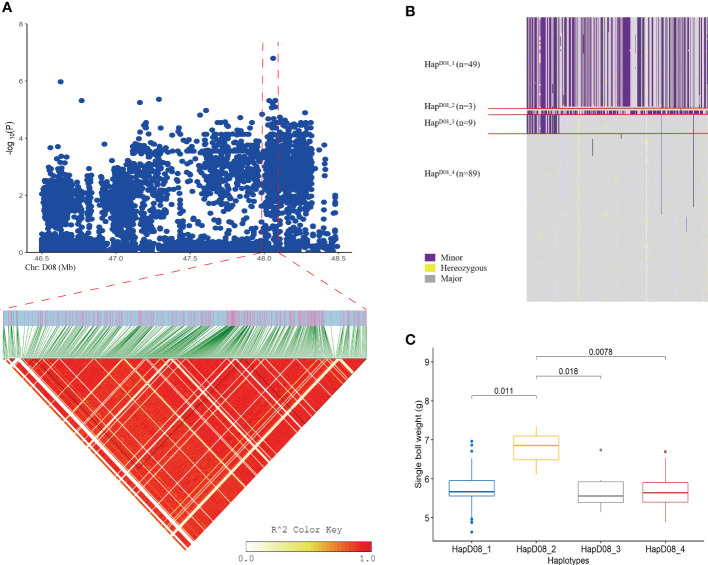
Loci related to the SBW trait found on chromosome D08. **(A)** Manhattan plot and LD block analysis of SBW from the GWAS; **(B)** Chr: D08:47.56-48.10 (Mb) interval haplotype analysis; **(C)** Difference analysis of EBN in different haplotypes.

To identify drought tolerance genes, we resequenced all the resource samples using upland cotton TM-1 (Yang et al., 2019) as the reference genome, and finally obtained 2,499,987 SNPs (the screening conditions were missing data <20% and MAF<1%). The highest density of SNPs was detected on chromosome A01, while the lowest density of SNPs was detected on chromosome A02, with an average marker density of 1.28 SNPs per kb (**Supplementary Table S5**). To explore the population structure characteristics and genotype structure of the tested upland cotton germplasm resources, we used ADMIX software. This analysis was based on the maximum likelihood estimation model and cross-validated for the number of subpopulations (k), thus the optimal number of ancestral components was determined (k=1-10). The results of the structural simulation analysis showed that when k=4, the CV error was minimized (**Figure 1A**). Therefore, a k value of 4 was selected to assess the genetic structure of the 150 cotton genotypes. In a principal component analysis (PCA) of these 150 cotton materials, 36.4% of the genetic variation was explained by the first two PCs (**Figure 2**). There was abundant genetic variation among the cotton varieties examined in this study. To further analyze the genetic differentiation of genotypes, NJ-based clustering was performed for the samples. Consistent with the ADMIX results, the stratified cluster tree showed significant differences among the variety complexes (**Figure 1C**). Four main clusters were defined in the tree; these groups corresponded to each of the major subgroups of the ADMIX analysis, which supports the division of the population into four major subgroups (**Figure 1B**). The corresponding Q matrix at k=4 was used for further marker-trait association mapping.

All identified high-quality SNP markers were used to estimate the degree of LD in the associated population. At the r^2^ = 0.428 threshold for all chromosomes, the average LD decay distance was approximately 500 kb (**Supplementary Figure S4**).

### 3.3 Genome-wide association analysis

**Figure 3:**
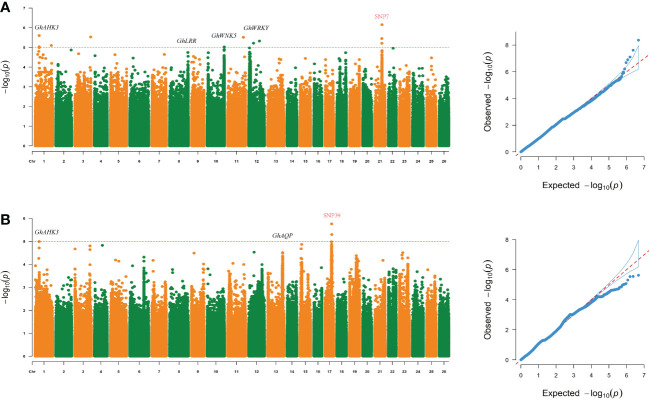
Loci related to the SBW and EFBN traits were found on chromosome 17 (chromosome D04) and chromosome 21 (chromosome D08) under drought stress. **(A, B)** Manhattan and QQ plots of GWAS results for SBW and EFBN.

To analyze and screen important genetic loci and candidate genes related to yield traits under drought stress conditions, different models were used for GWASs of 8 traits in each single environment and in multiple environments. The FarmCPU software program was used to analyze the associations between the screened SNP markers and the 8 traits in the 150 genotypes to detect marker-trait associations. SNP loci that were significantly associated with yield traits were identified under drought stress, and the loci were stable across environments (**Figure 3**).

### 3.4 Yield traits

#### 3.4.1 Control dataset

Under normal control conditions, 718 SNP markers were found to be significantly associated with the three yield traits, scattered across 26 chromosomes (**Supplementary Figure S5**). The Manhattan plot (**Supplementary Figure S5**) showed that of the 470 SNPs significantly associated with SBW, 9 were located on chromosome A03, 251 on chromosome A11, 191 on chromosome A12, and 19 on chromosome D06.

#### 3.4.2 Drought treatment dataset

Under drought stress, 126 significant SNPs associated with yield traits were identified. The Manhattan plot (**Figure 3**) showed that a total of 22 SNP markers above the threshold (**Supplementary Table S6**) that were associated with SBW were distributed on chromosome D08. The most significantly correlated SNP marker was ChrD08_48059786 (-log (*P*) =6.47). SNP4 (SNP D06_47161952) also had a high -log (*P*) value (5.52) (**Supplementary Table S6**).

Five important sites associated with SBW were identified. Importantly, SNP 7 (SNP D08_48059786) was located upstream of Gh_D08G143300, and a continuous signal was observed near this site in the Manhattan plot (**Figures 2**, **3** and **Supplemental Table S6**). We analyzed LD blocks in the 300 kb region upstream and downstream of this site and found that the SNP was closely related to block LD_SBW (D08: 47.56-48.10) (**Figure 2A** and **Supplementary Table S6**). Further haplotype analysis of this region revealed that all materials could be classified into four haplotypes and that HapD08_2, which was located in LD_SBW in this region, was associated with a higher SBW than the other three haplotypes (**Figures 2C**).

### 3.5 EFBN

**Figure 4:**
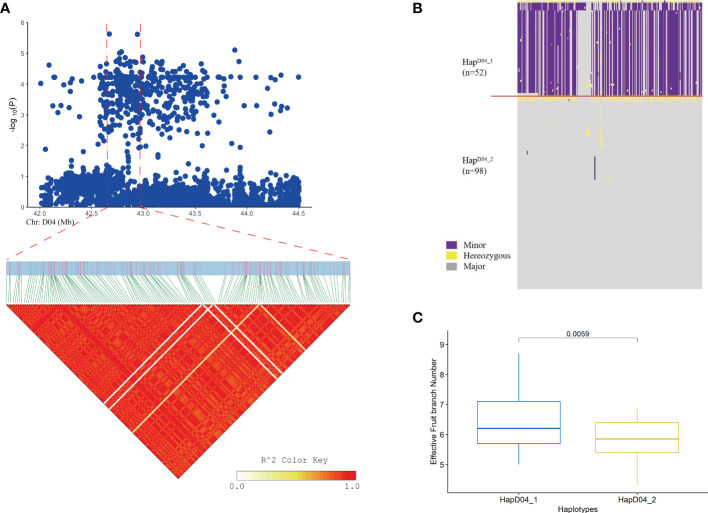
Loci related to the EFBN trait found on chromosome D04. **(A)** Manhattan plot and LD block analysis of EFBN from the GWAS; **(B)** Chr: D04.42.90-43.01 (Mb) interval haplotype analysis; **(C)** Difference analysis of EBN in different haplotypes.

A GWAS was performed for the EFBN phenotype in each environment. A total of nine important EFBN SNPs were identified under drought conditions in both environments, all of which were located on chromosome D04. Two SNPs significantly associated with EFBN were found on this chromosome, located at ChrD04_42670538 (SNP39, -log (*P*) =5.63) and ChrD04_42942695 (SNP45, -log (*P*) =5.55) (**Figure 4** and **Supplementary Table S6**). LD blocks in the 300 kb region upstream and downstream of this site were also analyzed, and LD block analysis showed that the peak SNP was mainly located in 42.90-43.01 Mb of chromosome D04 (**Figures 4A, B**). The haplotype analysis of this region showed that all materials could be divided into two haplotypes, and the HapD04_1 haplotype samples showed higher EFBN values than the HapD04_2 haplotype samples (**Figure 4C**).

### 3.6 EBN

A total of 77 SNPs were significantly associated with EBN. Eighteen of them were consecutive and located on chromosome D08. LD block analysis revealed that the peak SNP D08_48059786 was in the closely linked LD_EBN region (D08: 46.63-48.27 Mb) (**Supplementary Figure S6A** and **Supplementary Table S6**). Further haplotype analysis of LD_EBN led to the classification of four haplotypes. The difference analysis of four haplotypes in the LD_EBN region showed that the EBN of HapD08_2 was higher than that of the other three haplotypes (**Supplementary Figures S6B, C**).

### 3.7 PH

Eleven SNPs were found to be significantly associated with PH under drought stress (**Supplementary Table S6**). Among the significant SNPs, there was one consecutive SNP signal on chromosomes A03 and A05 in one environment in 2019. In another environment, there was a continuous SNP signal on chromosome A08 (**Supplementary Table S6**).

### 3.8 FBN

A GWAS of FBN in each environment revealed 91 SNPs significantly related to this trait. There were 9 significant SNPs on chromosome A13 in a single environment, and 7 significant SNPs on chromosome A13 under drought stress. Another continuous SNP signal was detected on chromosome D08 under drought stress, with a total of 19 significant SNP loci, 17 of which were on chromosome D08 (**Supplementary Table S6**).

### 3.9 BN

Fifty-nine SNPs were found to be significantly associated with BN (**Supplementary Table S6**). Under the control conditions in the two environments, continuous SNP signals were found on chromosome A07, with a total of 40 significant SNP sites, and the peak value of the SNPs was mainly distributed between 2.13-2.20 Mb on chromosome A07 (**Supplementary Table S6**).

### 3.10 Candidate gene screening and qRT-PCR expression analysis

**Figure 5:**
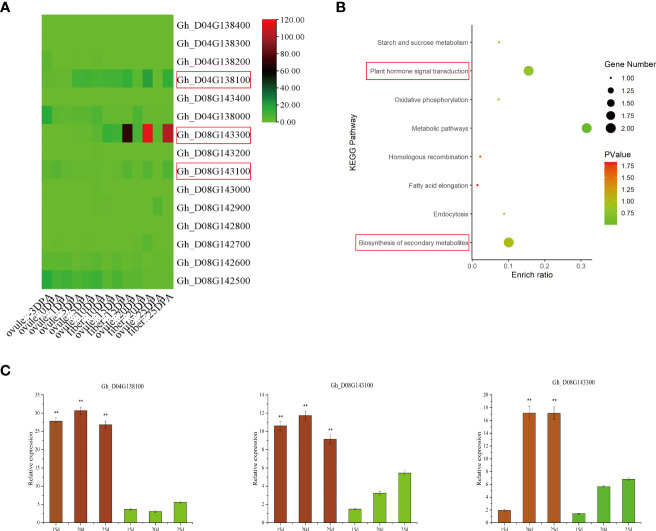
Transcriptome data and qRT-PCR analysis of 15 candidate genes. **(A)** Analysis of the transcriptome data of 15 candidate genes at 10, 15 and 20 DPA of ovule and fiber development. **(B)** KEGG analysis of 15 candidate genes. **(C)** qRT-PCR was used to analyze the expression of three genes at 10, 15 and 20 d of fiber development, and significant differences are indicated by ** (p < 0.01).

The LD decay distance can be used as the confidence interval of candidate genes, but due to the characteristics of the cotton genome, the LD decay distance is long. Therefore, in our analysis of the location of significant SNPs in the upland cotton genome, we searched within 500 kb-1 Mb on each side of significant SNP markers to analyze and identify candidate genes associated with drought tolerance traits. Gene functions associated with identified SNPs were assigned using the Universal Protein Database (UniProt) and the Cotton Genome Database (**Table 1**). In this study, the strongest signals identified on chromosomes D04 and D08 were novel, which resulted in the identification of 15 candidate genes within the candidate intervals on the chromosomes, including 5 on D04 and 10 on D08. Under drought stress, SBW, SC and EBN were mapped to the same region on chromosome D08, while EFBN, another trait significantly associated with yield, was mapped to a region located on chromosome D04 (**Figure 3**). Among the yield traits, 10 common genes were identified in the common interval of chromosome D08 (**Table 1**). Gene Ontology (GO) enrichment analysis showed that candidate genes in all ranges were significantly enriched in two functional categories (inorganic diphosphatase activity and phosphate-containing compound metabolic process) (**Supplementary Table S7**). Kyoto Encyclopedia of Genes and Genomes (KEGG) annotation revealed that the metabolic pathways of these candidate genes are closely related to the biosynthesis of secondary metabolites and plant hormone signal transduction (**Supplementary Table S8** and **Figure 5B**). These candidate genes are important because they are the candidate genes most likely to enhance cotton drought tolerance and mitigate yield loss under drought stress. To further reduce the number of candidate genes, according to published cotton RNA-seq data, we found that there were significant differences in the expression levels of 4 of the 12 functionally annotated genes between the control and drought stress treatments. All four of these genes showed upregulated expression after drought stress treatment; moreover, the expression trend of the remaining 8 genes after drought stress treatment was not obvious (**Supplementary Figure S7A**). In addition, these four genes were highly expressed in ovules and fibers, and three of the genes were significantly highly expressed at 10, 15 and 25 day psot anthesis (DPA) (**Figure 5A**).

**Table 1:** Candidate genes and their annotation of loci related to yield traits under drought stress in GWAS analysis.

Gene ID	Strand	Start	End	Annotation
Gh_D04G138000	–	42952898	42956108	Probable starch synthase 4, chloroplastic/amyloplastic
Gh_D04G138100	+	42971241	42973724	ADP-ribosylation factor GTPase-activating protein AGD7
Gh_D04G138200	–	42980192	42980500	Ribonuclease HI
Gh_D04G138300	–	42997896	42998282	Serine/threonine-protein phosphatase 7 long form homolog
Gh_D04G138400	–	42999850	43003930	Cation/H(+) antiporter 20
Gh_D08G142500	+	47579389	47583876	NAC domain-containing protein 69
Gh_D08G142600	+	47585542	47587607	Unkown
Gh_D08G142700	–	47587523	47589628	Soluble inorganic pyrophosphatase 1
Gh_D08G142800	–	47592151	47594025	3-ketoacyl-CoA synthase 1
Gh_D08G142900	–	47735907	47737840	Unkown
Gh_D08G143000	–	47766366	47767796	Transcription factor bHLH14
Gh_D08G143100	–	47954594	47957599	Rho GTPase-activating protein 3
Gh_D08G143200	–	47963917	47965509	Serine/threonine-protein phosphatase 7 long form homolog
Gh_D08G143300	–	47975232	47976089	Auxin-responsive protein SAUR32
Gh_D08G143400	+	48071810	48072772	Unkown

The positive and negative directions of the chain are chain are indicated by the plus sign + and the minus sign – respectively.

These three highly expressed candidate genes were selected to verify the RNA-seq data. The three candidate genes included one encoding auxin reactive protein (SAUR) and two activating protein GTPases (**Table 1**). To determine whether the expression of the three genes was induced by drought stress, one drought-tolerant material (Xinluzao 45) and one drought-sensitive material (Xinluzao 26) were selected to analyze the expression levels of the three genes after drought stress. qRT-PCR was used to detect the relative expression levels of these genes, and the value of the *GhUBQ7* gene was used as the threshold (internal control) for normalization. The results were then compared with the transcriptome results. These results showed that the genes were expressed differently in resistant lines after drought stress, and their expression patterns were basically consistent with the RNA-seq data. The qRT-PCR results for these genes showed significantly higher expression in the drought-tolerant materials than in the drought-sensitive materials (**Supplementary Figure S7B**). In addition, at 10, 15 and 20 d of fiber development, the expression levels of these three genes in the two materials with different drought tolerance levels were analyzed by qRT-PCR. The results showed that the expression of these three genes in the materials with strong drought tolerance was higher than that in the drought sensitive material (**Figure 5C**). Therefore, these three genes are significantly related to the drought tolerance of cotton, suggesting their role as candidate drought tolerance genes related to yield traits of cotton under drought.

## 4 Discussion

Eight yield-related traits of 150 upland cotton germplasms were analyzed by the GWAS approach. In addition, the leaf tissue and fibrous tissue of two materials with different levels of drought resistance were collected and used for qRT-PCR analysis. The results of this study add to the understanding of the variation in yield traits under drought stress. The results can provide a reference for the improvement of cotton molecular breeding under drought conditions.

In all the tested materials, yield traits and other traits were significantly different between the control and drought conditions, indicating a large amount of genetic variation in drought tolerance among the materials. Phenotypic analysis showed that drought treatment significantly affected the traits of the different materials (**Supplementary Table S3**). SBW, EFBN and EBN were all significantly positively correlated under drought stress, indicating that improving these traits at the same time would lead to an increase in SC yield (**Supplementary Table S4**) (Sun et al., 2018). The population structure in all the tested materials was analyzed according to the K value, and the population was divided into four categories (**Figure 1B**), indicating some variation within the population. Phylogenetic analyses showed similar results (**Figure 1C**), indicating that these analyses can play a role in preventing false positives in GWASs (Soto-Cerda and Cloutier, 2012; Eltaher et al., 2018). The genome-wide LD decayed to half the r^2^ (0.428) at 500 kb, and there were a large number of significant SNP markers in LD, suggesting that significant marker-trait association can be found using a GWAS (Park et al., 2008; Schwarz et al., 2015). The population structure shown in the analysis results of the Q-Q diagram is well explained because most of the points are on the diagonal for all traits (**Figure 3**) (Burghardt et al., 2017; Paterne et al., 2021). Cotton yield is a complex quantitative trait that is greatly affected by the environment. Although cotton resources are abundant, due to the large genome of cotton, the yield traits of cotton have not been fully explored, especially under drought conditions (Sun et al., 2018; Said et al., 2015). Yield traits can indirectly reflect the drought tolerance of cotton (Sun et al., 2021), among which SBW is an important trait related to yield, and EBN is another trait with a significant contribution to yield per plant (Sun et al., 2018; Sun et al., 2021). The results of this study showed that under drought stress, SBW and EBN were stably associated with SNPs on chromosome D08 in both environments, and there were 29 significant SNPs related to SBW and EBN (**Figures 3**, **2**; **Supplementary Figure S6A**) (Wang et al., 2019), which were different from those located on A07, D03, D06, D09 and D12 (Chen et al., 2008; Ma et al., 2008; Wu et al., 2009; Vollmer et al., 2011; Ning et al., 2013; Yu et al., 2013; Sun et al., 2018). However, Fang et al. identified a significant SNP related to BN that was located adjacent to D08, and there were only three genes in the LD block of this site. One of the genes was differentially expressed in the ovule and fiber of the two different materials, and the haplotype analysis verified this result (Fang et al., 2017). However, the SNP site on chromosome D08 identified in this study is a novel locus associated with yield under drought conditions.

In this study, we identified three candidate genes by GWAS that were supported by published RNA-seq data, one of which is *Gh_D08G143300*, a homolog of Arabidopsis *SAUR32* (Ren and Gray, 2015; Stortenbeker and Bemer, 2019; Zhou et al., 2022). The other two genes are *Gh_D08G143100*, which is homologous to Arabidopsis *ROPGAP3*, and *Gh_D04G138100*, which is homologous to Arabidopsis *AGD7* (Myung et al., 2007; Yoshihisa and Hiroo, 2012). Therefore, *Gh_D08G143300* may also affect auxin synthesis and transport in the fiber under drought stress, leading to the redistribution of auxin and thus promoting the growth of cotton fiber. However, *Gh_D08G143100* may alternatively initiate a unique pattern in the secondary cell wall of fibers under drought stress. *Gh_D04G138100* is activated under drought stress and may be involved in protein transport. The RNA-seq and qRT-PCR results showed that the three candidate genes were differentially expressed in the materials with large differences in drought tolerance (**Figure 5** and **Supplementary Figure S7**). These results suggest that these three candidate genes may be one of the important genes in determining cotton yield formation under drought stress. More studies are needed to further analyze and verify how these three genes affect cotton yield under drought stress and to verify their functions in yield formation under such conditions.

## 5 Conclusion

To explore the regulatory mechanism of cotton yield variation under drought stress, 150 upland cotton germplasms were selected, and GWAS was conducted on three yield traits (SBW, SC and LC) and five agronomic traits (related to plant height and fruit branch number) that are closely related to yield. The GWAS results revealed a total of 46 significant SNPs under drought stress, and 15 candidate genes were screened. Three differentially expressed genes (*Gh_D04G138100*, *Gh_D08G143100* and *Gh_D08G143300*) were screened by combining published RNA-seq data. Two materials with extreme drought resistance differences, Xinluzao 45 and Xinluzao 26, were selected, and these two materials also showed significant differences in drought resistance in the field experiment. qRT-PCR was used to verify the expression patterns of the three candidate genes after drought stress in the two materials with drought resistance extremes. The results showed that high expression of these genes was induced by drought stress. At the same time, there were significant differences in the expression of these three genes in the developed fibers. In this paper, we further analyzed the molecular markers and candidate genes related to upland cotton yield under drought stress, and the findings will be helpful for studying the molecular mechanism of cotton yield traits under drought stress.

## Data availability statement

The data presented in the study are deposited in the NCBI repository, accession number PRJNA 605345.

## Author contributions

FS analyzed the data and drafted the manuscript. JM and YY provided ideas, designed and supervised the experiment. JM and WS provided cotton seeds, and all authors reviewed the manuscript. All authors contributed to the article and approved the submitted version.
