# Supplementary material for: Genome-wide association analysis revealed genetic variation and candidate genes associated with the yield traits of upland cotton under drought conditions
Source: Front Plant Sci. 2023 Apr 12;14:1135302. doi: 10.3389/fpls.2023.1135302 (PMC10130383; doi:10.3389/fpls.2023.1135302)
Supplement: Supplementary file 1 [file DataSheet_1.docx]

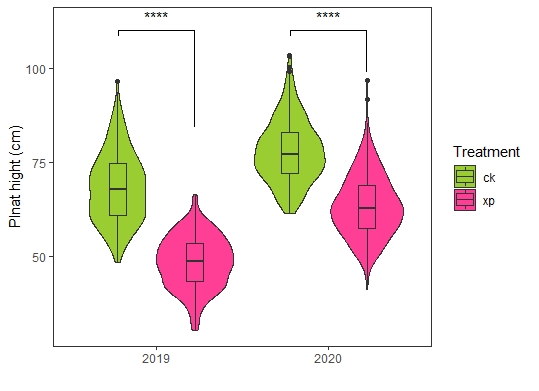

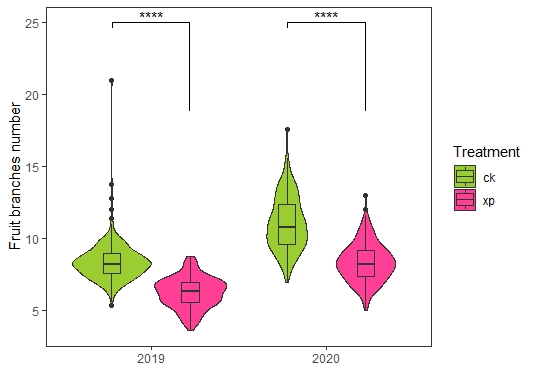


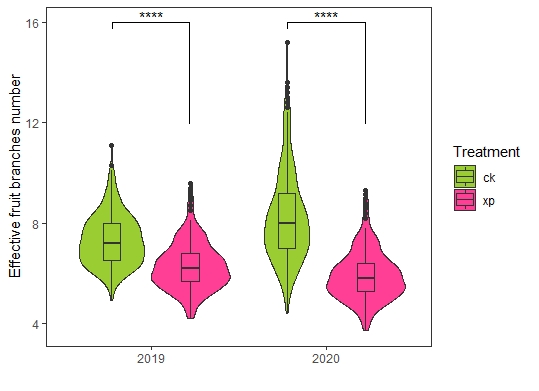

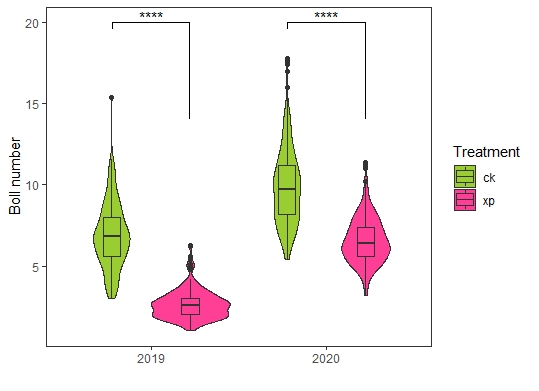


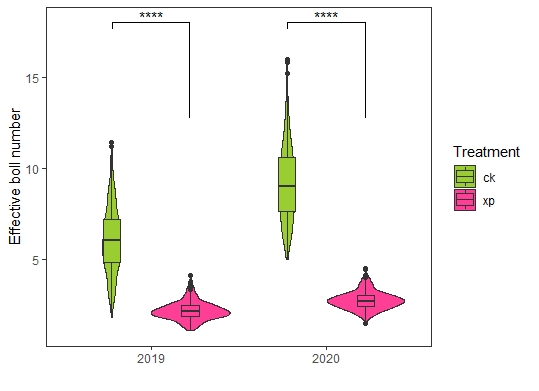

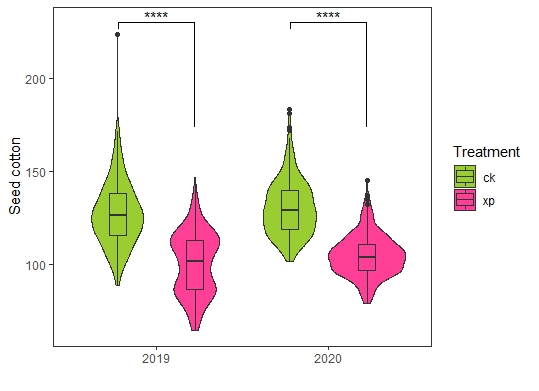


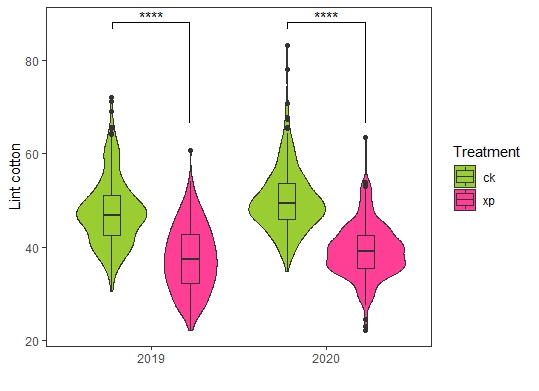

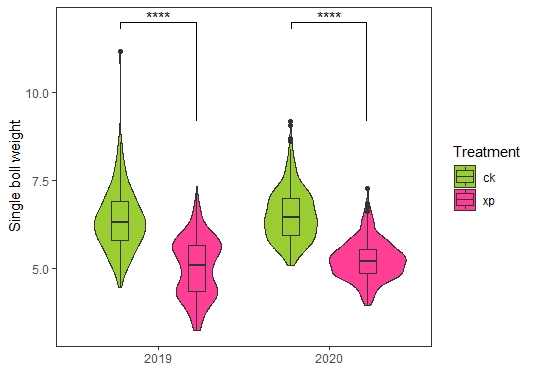


Supplementary Figure S1 Difference analysis of three yield traits and five agronomic traits in two years (ck: normal control, xp: drought stress treatment)


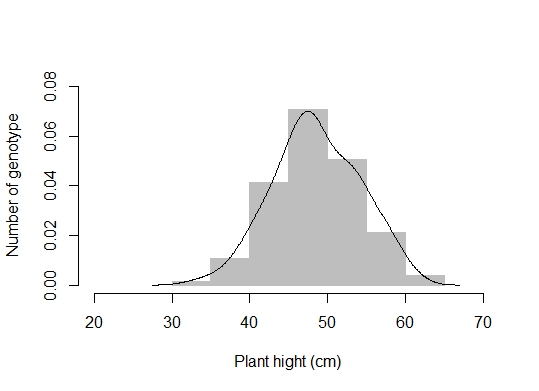

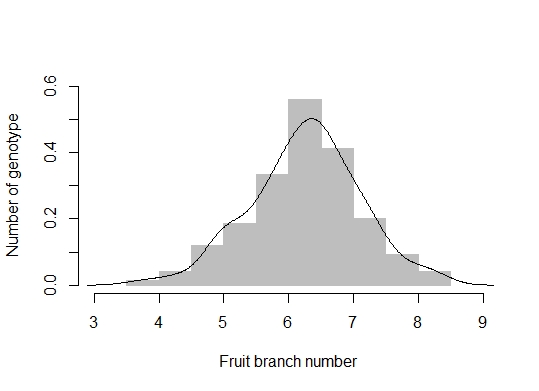


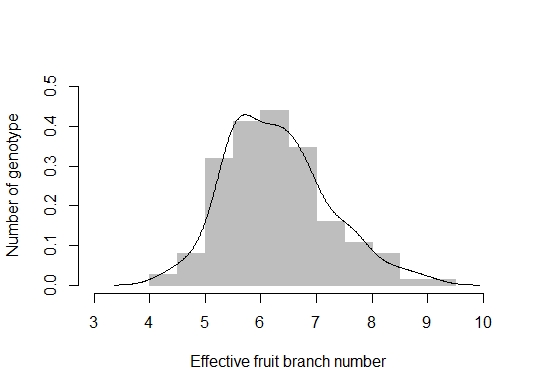

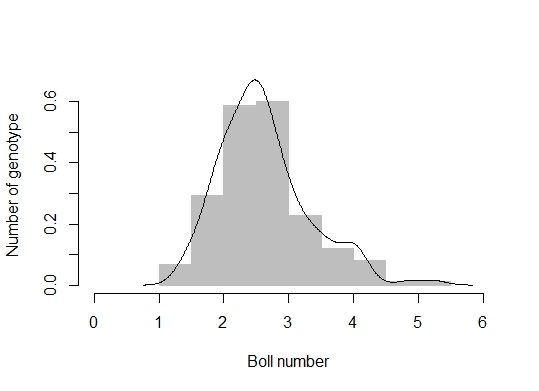


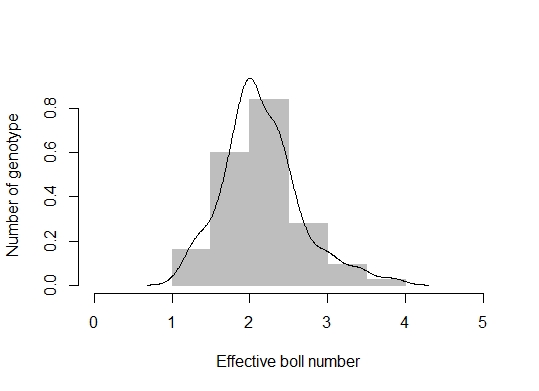

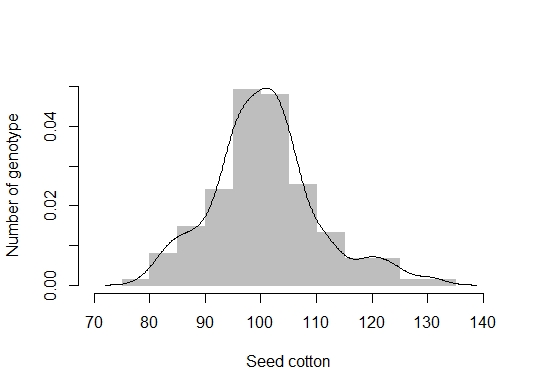

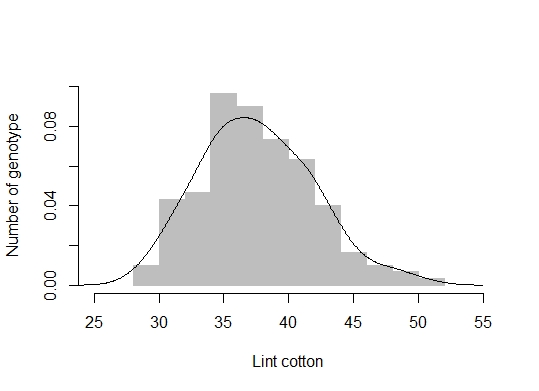

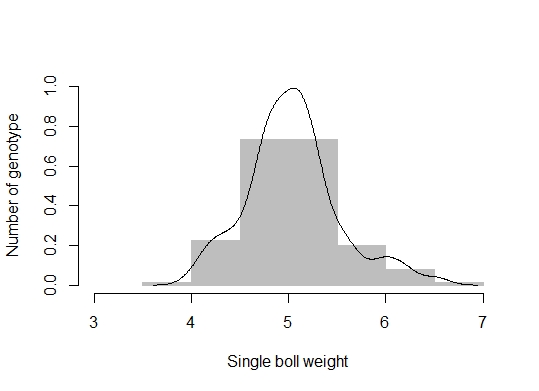


Supplementary Figure S2 Histogram of the frequency distribution of 8 traits


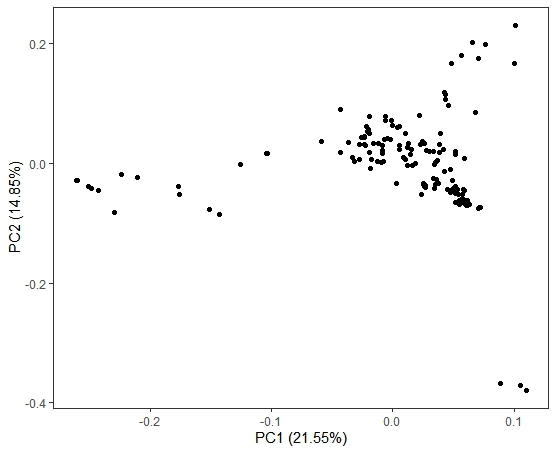


Supplementary Figure S3 PCA plot of the first two components (PC1 and PC2)


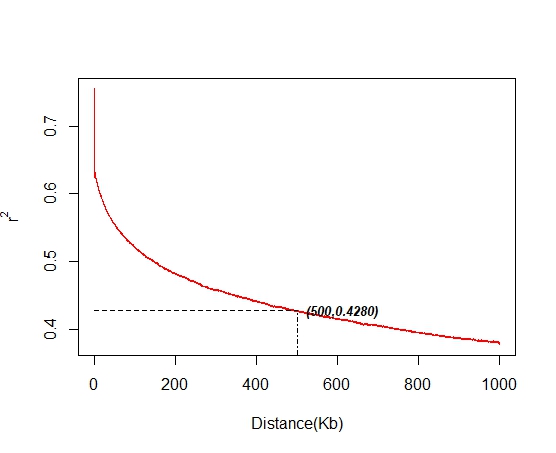


Supplementary Figure S4 Linkage disequilibrium (LD) of the tested germplasm


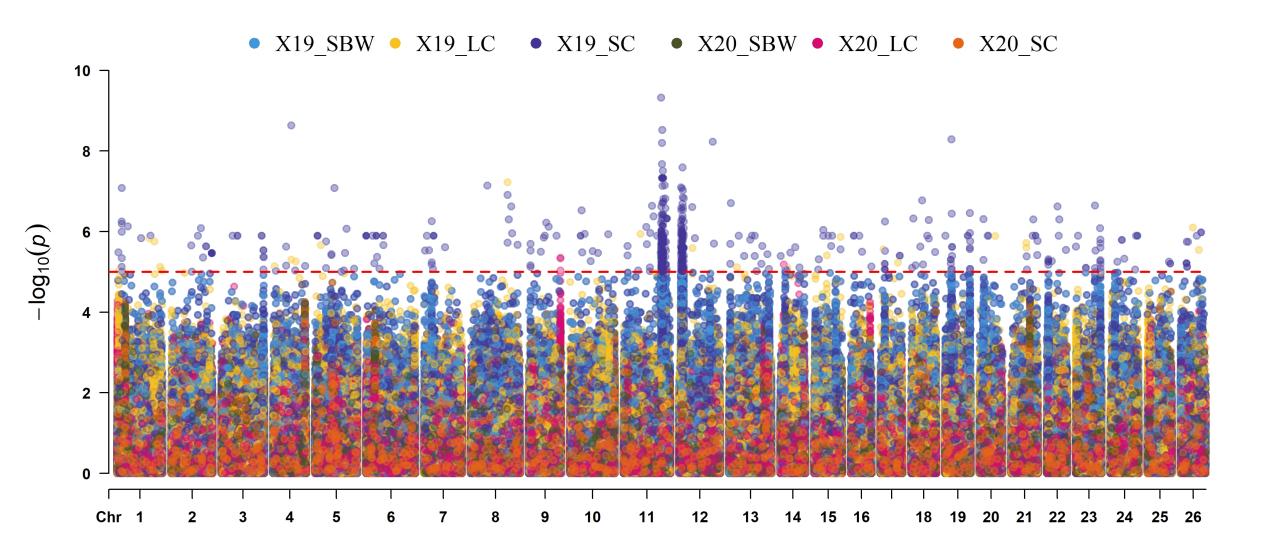


Supplementary Figure S5 Manhattan distribution map of three yield traits under normal conditions.


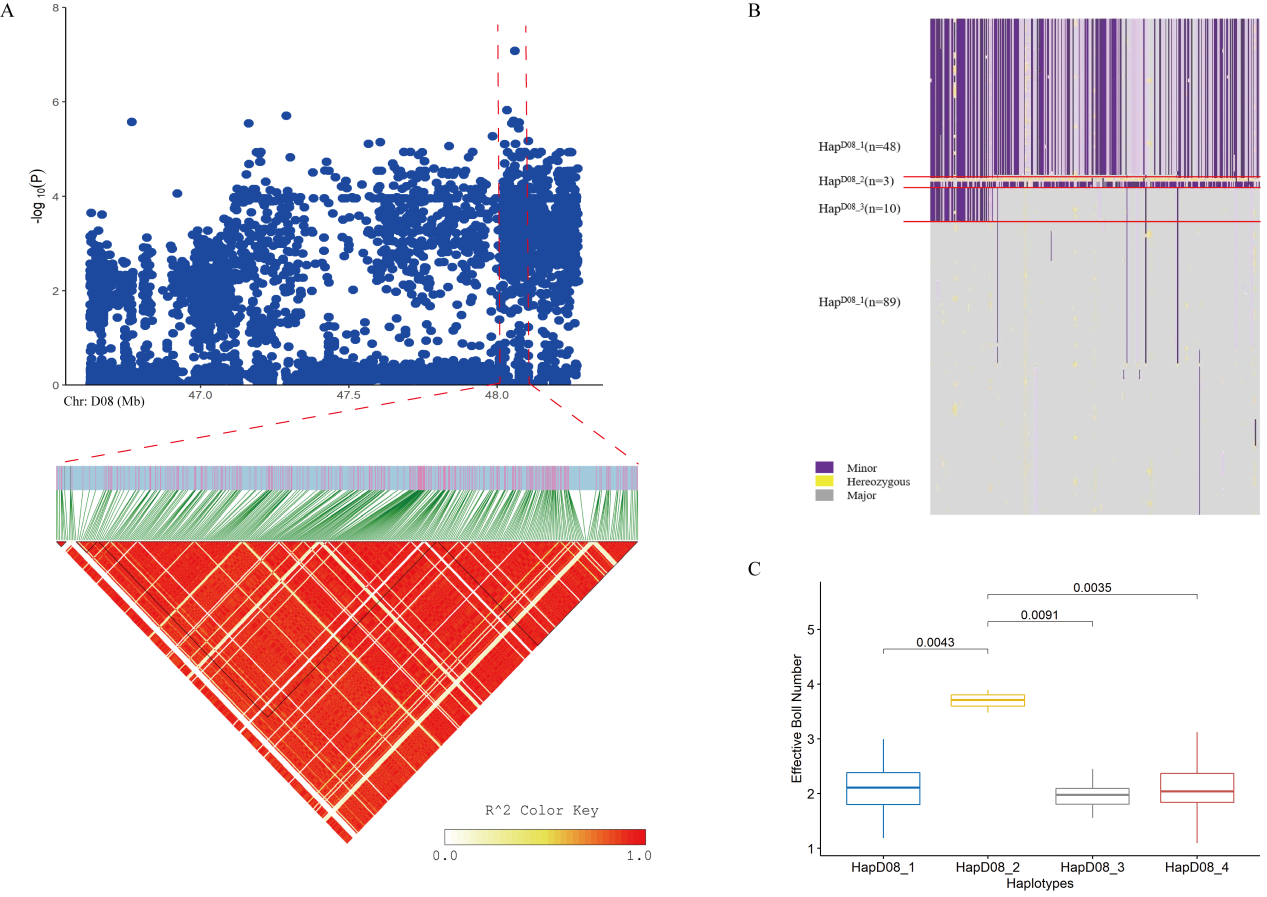


Supplementary Figure S6 EBN related loci were found on chromosome D08. A: EBN GWAS Manhattan and LD block analysis; B: chrD08:46.63-48.27 (Mb) interval haplotype analysis; C: Difference analysis of EBN in different haplotypes.


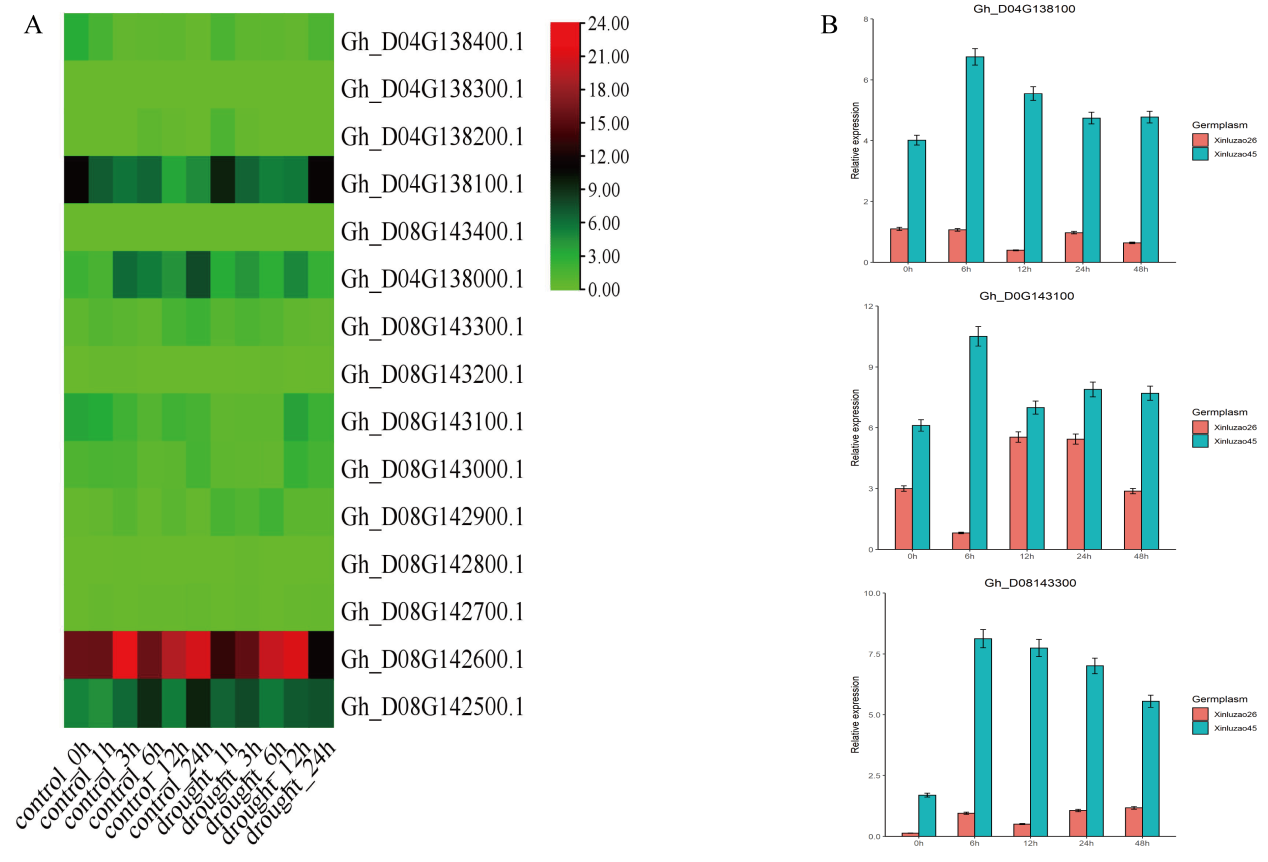


Supplementary Figure S7 A: Transcriptional expression analysis of 15 candidate genes. B: qRT-PCR analysis of the three candidate genes after 48 h of stress showed significant differences in ** (p < 0.01).
